# Prevalence of Thyroid Dysfunction in Patients With Hepatitis C

**DOI:** 10.7759/cureus.18289

**Published:** 2021-09-26

**Authors:** Kefayatullah Nazary, Sana Anwar, Ankita Y Choudhary, Deepa Malla, Farukhzad Hafizyar, Abdul Subhan Talpur, Faryal Fatima, Marjan Khan

**Affiliations:** 1 Internal Medicine, Kabul University of Medical Sciences, Kabul, AFG; 2 Internal Medicine, Lugansk State Medical University, Luhansk, UKR; 3 Integrative Medicine, Dr. NTR University of Health Sciences, Hyderabad, IND; 4 Internal Medicine, Patan Hospital, Kathmandu, NPL; 5 Internal Medicine, Ariana Sabet Hospital, Kabul, AFG; 6 Medicine, Liaquat University of Medical and Health Sciences, Jamshoro, PAK; 7 Internal Medicine, Marshfield Clinic Health System, Marshfield, USA

**Keywords:** hepatitis-c infection, association, autoimmune, thyroid dysfunction, hepatitis c

## Abstract

Introduction: Hepatitis C has been linked to a multitude of autoimmune disorders, including rheumatoid arthritis, thyroid disease, cryoglobulinemia, immune thrombocytopenic purpura, systemic lupus erythematosus, and Sjögren’s syndrome. In this study, efforts were made to draw a parallel between hepatitis C and thyroid dysfunction.

Methods: This case-control study was conducted between June 2020 and March 2021 in the gastroenterology ward of a tertiary care hospital. We enrolled 300 hepatitis C-positive patients in this study through consecutive convenient non-probability sampling. In addition, 300 patients without hepatitis C were signed up as a control group. Blood sampling for thyroid function tests was conducted via phlebotomy from the cubital vein and the samples were dispatched to the laboratory for further study.

Results: The control group had more euthyroid patients as compared to patients with hepatitis C (74.6% vs. 89.6%; p-value: <0.01). Hepatitis C patients had more cases of primary hypothyroidism compared to the control group (10.6% vs. 4.6%; p-value: 0.005). Similarly, patients with hepatitis C had a higher prevalence of subclinical hypothyroidism compared to the control group (6.0% vs. 1.3%; p-value: 0.002).

Conclusion: Hepatitis C patients have a high frequency of thyroid dysfunction, particularly primary hypothyroidism and subclinical hypothyroidism. Therefore, it is important to ensure regular screening for early prognosis and avoid treatment modalities that are known to cause thyroid abnormalities.

## Introduction

In the light of previously researched data, the hepatitis C virus has been estimated to cause 15-20% cases of acute hepatitis, followed by roughly 50-80% cases of chronic infection [1]. Furthermore, chronic hepatitis C patients will go on to develop fatal complications like cirrhosis in around 20% of cases and hepatocellular carcinoma (HCC) at a rate of 4-5% per year in cirrhotic patients [2-4].

In addition to the dangers of developing high-risk complications, hepatitis C has also been incriminated with a wide spectrum of autoimmune disorders, including rheumatoid arthritis, thyroid disease, cryoglobulinemia, immune thrombocytopenic purpura, systemic lupus erythematosus, and Sjögren’s syndrome [5]. A study by Antonelli et al. established that thyroid autoimmune diseases were far more common in patients with chronic hepatitis C, even in the absence of cirrhosis, HCC, or interferon treatment in comparison to the normal controls [6].

During our extensive literature review, we came to the conclusion that there is a lack of data, drawing out the relation between hepatitis C and thyroid abnormalities, particularly in the South Asian region. Worldwide, Pakistan accounts for the second most number of cases of hepatitis C, with a prevalence of 4.8% [7]. However, there is a scarcity of data reported about the association of hepatitis C with other autoimmune disorders, especially thyroid disorders. In this study, efforts were made to bridge this gap by determining the frequency of thyroid dysfunction in patients with hepatitis C.

## Materials and methods

This case-control study was conducted between June 2020 and March 2021 in the gastroenterology ward of a tertiary care hospital. We enrolled 300 treatment-naive hepatitis C-positive patients in the study through consecutive convenient non-probability sampling. Ethical review board approval was taken from Liaquat University of Medical and Health Sciences (LUMHS/IRB-OFC/2020-06-07). The presence of hepatitis C infection was established via polymerase chain reaction (PCR). Another 300 patients without hepatitis C were signed up as a control group. However, patients who have had a prior diagnosis of thyroid disease, or were on thyroid medications, or had undergone thyroid surgery were omitted from this study.

After determining a group of patients to be studied, blood sampling for thyroid function tests was conducted via phlebotomy from the cubital vein and the samples were dispatched to the laboratory for further investigations.

Serum free thyroxine (FT4) was determined by radioimmunoassay (RIA) and thyroid-stimulating hormone (TSH) was evaluated by immunoradiometric assay (IRMA) techniques, using commercial kits of Immunotech Inc. (Beckman, Czech Republic). Normal ranges for FT4, free triiodothyronine (FT3), and TSH, as standardized in our laboratory, are 11.0-22.0 pmol/L, 2.5-5.8 pmol/L, and 0.3-4.0 mIU/L, respectively. Thyroid dysfunction was classified based on Table 1.

**Table 1:** Levels of T4 and TSH in thyroid dysfunction. T4: thyroxine; TSH: thyroid-stimulating hormone.

Thyroid dysfunction	T4	TSH
Primary hyperthyroidism	Increased	Decreased
Primary hypothyroidism	Decreased	Increased
Subclinical hypothyroidism	Normal	Increased
Subclinical hyperthyroidism	Normal	Decreased

Statistical analysis was conducted with the help of Statistical Package for the Social Sciences (SPSS v. 23.0) (IBM Corp., Armonk, New York). Numerical data were presented as mean and standard deviation, while categorical data were presented as frequency and percentages. Unpaired t-test and chi-square test were used to compare case and control groups. A p-value lower than 0.05 meant that the difference between the intervention and control group was significant and the null hypothesis was not valid.

## Results

The age of participants in the hepatitis C group was 41 ± 10 years and in the control group was 39 ± 11 years; however, the difference was non-significant. Other parameters were also comparable between the two groups (Table 2).

**Table 2:** Comparison of characteristics of both groups. FT4: free thyroxine; mIU/L: milli-international units per liter; NS: nonsignificant; pmol/L: picomoles per liter; TSH: thyroid-stimulating hormone.

Characteristics	Case group	Control group	p-value
Age in years	41 ± 10	39 ± 11	NS
Gender			
Male	171 (57.0%)	168 (56.0%)	NS
Female	129 (43.0%)	132 (44.0%)	NS
Mean values			
FT4 (pmol/L)	17.7 ± 4.6	18.2 ± 4.2	NS
TSH (mIU/L)	2.1 ± 0.8	1.9 ± 0.6	NS

The control group had more euthyroid patients as compared to patients with hepatitis C (74.6% vs. 89.6%; OR: 0.33; p-value < 0.01). Hepatitis C patients had more cases of primary hypothyroidism compared to the control group (10.6% vs. 4.6%; OR: 2.43; p-value: 0.0072). Similarly, patients with hepatitis C had a higher prevalence of subclinical hypothyroidism compared to the control group (6.0% vs. 1.3%; OR: 4.72; p-value: 0.002) (Table 3).

**Table 3:** Thyroid dysfunction in case and control groups. CI: confidence interval.

Thyroid dysfunction	Case group	Control group	Odds ratio (95% CI)	p-value
Euthyroid	224 (74.6%)	269 (89.6%)	0.33 (0.21-0.53)	<0.01
Primary hyperthyroidism	18 (6.0%)	09 (3.0%)	2.06 (0.91-4.67)	0.07
Primary hypothyroidism	32 (10.6%)	14 (4.6%)	2.43 (1.27-4.67)	0.0072
Subclinical hyperthyroidism	08 (2.6%)	04 (1.3%)	2.02 (0.60-6.80)	0.24
Subclinical hypothyroidism	18 (6.0%)	04 (1.3%)	4.72 (1.57-14.12)	0.005

## Discussion

Our study states that the prevalence of euthyroidism was significantly lower in patients with hepatitis C compared to the control group. Moreover, hepatitis C was observed to be linked with primary and subclinical hypothyroidism more than the control group. Supporting the results of our study, there are few studies in the literature proving the link between hepatitis C infection and primary hypothyroidism, which was observed to be the most common thyroid abnormality in our study as well.

Several studies have also proved the association between hepatitis C infection and autoimmune thyroiditis (AT) [8-10]. A study conducted by Ganne-Carrie et al. demonstrated a higher prevalence of AT in chronic hepatitis C patients; the frequency of thyroid abnormalities was more in hepatitis C patients than those who did not have the infection [9]. This association is explained by the phenomenon that the various antigens of the hepatitis C virus could potentially lead to the development of thyroid autoimmune disease [11] because of mechanisms like duplication of its molecules [12]. Another hypothesis believed states that hepatitis C could not specifically cause AT but triggers the production of non-organ-specific autoantibodies (NOSAs) [13].

Thyroid abnormalities in chronic hepatitis C patients could be due to an autoimmunity-related phenomenon. Even if the patients do not undergo treatment with interferon-alpha (IFN-α) and ribavirin, thyroid autoantibodies such as anti-thyroperoxidase (TPO) and anti-thyroglobulin antibodies (TGA) are seen [14]. Due to viral infection, the innate immune system helps form endogenous IFN-α and interferon-beta (IFN-β) in the thyroid gland. Endogenous and exogenous interferons (IFN), in turn, form thyroid antibodies by natural killer cells and memory T cell activation [14]. Moreover, patients treated with IFN-α and ribavirin are shown to report thyroid abnormalities like hypothyroidism, hyperthyroidism, and thyroiditis [15-17]. Treatment with pegylated IFN with ribavirin could cause hypophysitis in chronic hepatitis C patients, leading to central hypothyroidism [14,15]. The IFN-α molecule and ribavirin have immunomodulating properties and directly affect the thyroid gland, inducing a toxic effect and/or autoimmune mechanism, i.e. the induction of TSH receptor autoantibodies, antithyroid autoantibodies, thyroid cell apoptosis, cell-mediated immunity, expression of major histocompatibility complex, and cytokine production regulation [14,15]. Therefore, due to these complications and adverse events, treatment options for hepatitis C should be chosen in a way that the thyroid is not affected.

The findings of our study point toward the fact that in patients with hepatitis C, thyroid abnormalities should be screened to avoid any complications. However, this study has certain limitations. It is a single-center, case-control study and future longitudinal studies covering multiple centers should be conducted to confirm our results. Our study did not determine the impact of the presence of thyroid dysfunction in the treatment of hepatitis C.

## Conclusions

Evidence shows that hepatitis C patients are more frequently seen to have problems related to thyroid, most commonly primary and subclinical hypothyroidism. Therefore, these patients should be screened at regular intervals for early prognosis. Treatment modalities that are known to cause thyroid abnormalities should be avoided in such patients.
